# Global-Scale Structure of the Eelgrass Microbiome

**DOI:** 10.1128/AEM.03391-16

**Published:** 2017-05-31

**Authors:** Ashkaan K. Fahimipour, Melissa R. Kardish, Jenna M. Lang, Jessica L. Green, Jonathan A. Eisen, John J. Stachowicz

**Affiliations:** a Institute of Ecology and Evolution, University of Oregon, Eugene, Oregon, USA; b Department of Evolution and Ecology, University of California, Davis, Davis, California, USA; c Genome Center, University of California, Davis, Davis, California, USA; d Department of Medical Microbiology and Immunology, University of California, Davis, Davis, California, USA; e Santa Fe Institute, Santa Fe, New Mexico, USA; University of Bayreuth

**Keywords:** phyllosphere-inhabiting microbes, plant-microbe interactions, rhizosphere-inhabiting microbes, seagrass

## Abstract

Plant-associated microorganisms are essential for their hosts' survival and performance. Yet, most plant microbiome studies to date have focused on terrestrial species sampled across relatively small spatial scales. Here, we report the results of a global-scale analysis of microbial communities associated with leaf and root surfaces of the marine eelgrass Zostera marina throughout its range in the Northern Hemisphere. By contrasting host microbiomes with those of surrounding seawater and sediment, we uncovered the structure, composition, and variability of microbial communities associated with eelgrass. We also investigated hypotheses about the assembly of the eelgrass microbiome using a metabolic modeling approach. Our results reveal leaf communities displaying high variability and spatial turnover that mirror their adjacent coastal seawater microbiomes. By contrast, roots showed relatively low compositional turnover and were distinct from surrounding sediment communities, a result driven by the enrichment of predicted sulfur-oxidizing bacterial taxa on root surfaces. Predictions from metabolic modeling of enriched taxa were consistent with a habitat-filtering community assembly mechanism whereby similarity in resource use drives taxonomic cooccurrence patterns on belowground, but not aboveground, host tissues. Our work provides evidence for a core eelgrass root microbiome with putative functional roles and highlights potentially disparate processes influencing microbial community assembly on different plant compartments.

**IMPORTANCE** Plants depend critically on their associated microbiome, yet the structure of microbial communities found on marine plants remains poorly understood in comparison to that for terrestrial species. Seagrasses are the only flowering plants that live entirely in marine environments. The return of terrestrial seagrass ancestors to oceans is among the most extreme habitat shifts documented in plants, making them an ideal testbed for the study of microbial symbioses with plants that experience relatively harsh abiotic conditions. In this study, we report the results of a global sampling effort to extensively characterize the structure of microbial communities associated with the widespread seagrass species Zostera marina, or eelgrass, across its geographic range. Our results reveal major differences in the structure and composition of above- versus belowground microbial communities on eelgrass surfaces, as well as their relationships with the environment and host.

## INTRODUCTION

The health and performance of plants are often modulated by their associated microbiomes. The colonization of above- and belowground plant tissues by microorganisms from surrounding environments initiates interactions that are essential for plant productivity (1, 2), fitness (3, 4), and disease resistance (5–7). The drivers of plant microbiome structure and composition and the ways in which plant hosts acquire microorganisms from surrounding microbial species pools therefore have consequences for ecosystem dynamics, biodiversity, and agricultural productivity (8–11). Recent studies have identified critical associations between host and environmental factors and patterns of microbial community structure on plant compartments such as leaves (e.g., see references 12 and 13) and roots (e.g., see references 6 and 14). Yet, most plant microbiome studies to date have focused on terrestrial species (10, 13, 15), while patterns in the structure and composition of microbial communities associated with marine plants remain poorly understood by comparison.

Seagrasses are the only flowering plants that live entirely in a marine environment. One widespread species, Zostera marina, or eelgrass, in particular, provides a habitat for ecologically diverse and economically important ecosystems along coasts throughout much of the Northern Hemisphere (16, 17). The return of terrestrial seagrass ancestors to oceans is among the most severe habitat shifts accomplished by vascular plants (18) and has prompted detailed study of the physiological adaptations associated with this shift (19, 20), including the tolerance of salinity and anoxic sediment conditions. Therefore, Z. marina is an ideal testbed for the study of microbial symbioses with plant hosts that uniquely exploit relatively harsh environments. Given that human activities are changing the nutrient conditions in habitats worldwide (21) and the central role of microorganisms in plant nutrition (10, 11, 15), there is a pressing need to answer basic empirical questions about microbial associates of plants such as seagrasses that experience atypical abiotic conditions, including questions about their geographic distributions, putative community assembly patterns, and functional roles.

Much of our current knowledge of seagrass symbionts comes from targeted surveys of specific bacterial taxa using culture-dependent methods and microscopy under laboratory conditions or from field studies at local or regional spatial scales (e.g., see references 22–24). These studies have generated hypotheses about key symbioses between seagrasses and their associated microorganisms owing to processes such as nitrogen fixation and sulfide detoxification by bacteria (24) and to the competition between microbes for host-supplied metabolites (23) on plant surfaces. While culture-independent techniques have been used to describe the microbiome composition in seagrass-colonized marine sediments (25–27), an extensive characterization of *in situ* seagrass leaf and root surface microbiomes across the host's geographic range is still lacking, leaving potentially important but unculturable microorganisms overlooked and making it difficult to identify general patterns in seagrass symbiont community structure, taxonomic cooccurrence, and community assembly.

**FIG 1:**
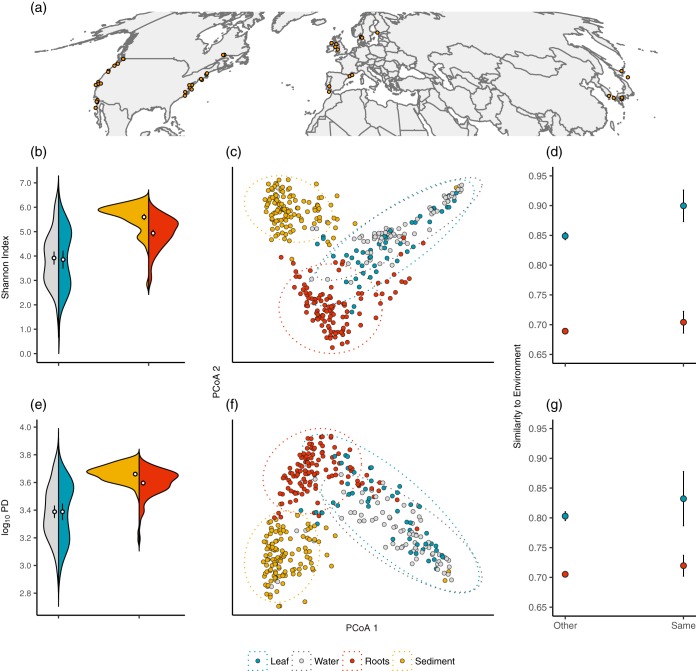
(a) Map of sampled seagrass beds. Orange points identify ZEN site coordinates. (b) Split violin plots showing means (white points) ± 2 (standard error of the mean [SEM]) values of rarefied Shannon diversity for leaf, water, root, and sediment microbial communities, where kernel density plots are shown on each side. (c) Ordination plot shows the results of an unconstrained principal-coordinate analysis (PCoA) of Canberra distances. Points represent microbial communities that are colored by their corresponding community type: blue, leaf; silver, seawater; red, root; and gold, sediment communities. Ellipses represent group-specific 95% confidence intervals assuming a multivariate *t* distribution. (d) Comparisons of host-environment compositional similarities within versus between seagrass beds. Points represent mean similarities between leaves and water (blue points) and roots and sediment (red points) ± 2 SEM. A significant relationship between host and environmental community similarities is seen only for leaves (*P* = 0.005). (e) Split violin plots showing means (white points) ± 2 SEM values of Faith's PD for the four microbial community types. (f) PCoA plot of unweighted UniFrac distances. (g) Comparisons of host-environment phylogenetic similarities within versus between seagrass beds. Points represent mean similarities between leaves and water (blue points) and roots and sediment (red points) ± 2 SEM.

Here, we report the results of a comprehensive analysis of microbial communities associated with leaf and root surfaces of individual Z. marina plants spanning their geographic range throughout the Northern Hemisphere. Namely, we sampled microbial communities present on the leaf and root surfaces of 129 eelgrass individuals (Fig. 1a) using the Illumina MiSeq platform to sequence amplified fragments of the V4 region of the 16S rRNA gene, which primarily targets environmental bacteria and archaea. To determine the relative contributions of potential microbial colonization sources, we also characterized the surrounding environments by sampling seawater and sediment microbial communities adjacent to each collected eelgrass host. We aimed to define the global structure, composition, and variability of symbiont communities associated with Z. marina, to contrast these communities with those of their surrounding seawater and sediment environments, and to generate new hypotheses about the mechanisms underlying the assembly of the eelgrass microbiome based on predictions from genome-scale metabolic modeling (28).

## RESULTS

We identified 23,285 unique microbial operational taxonomic units (OTUs) on eelgrass host surfaces. Bacterial community α-diversity analyses revealed a higher taxonomic diversity (Fig. 1b) on belowground root tissues compared with the microbial communities detected on leaves (linear mixed effects [LME] analysis of Shannon diversity; *F*_3,311_ = 100.76, *P* < 0.001) at the rarefaction depth considered herein. In contrast to belowground sediment communities, which exhibited higher taxonomic diversities than root communities on average (Tukey's *post hoc* test; *P* < 0.001), we did not detect a difference in Shannon diversity between eelgrass leaf and seawater microbial communities (*P* = 0.838) (Fig. 1b). A larger fraction of the taxa detected on leaves were rare than those on roots—92.5% of the OTUs detected on leaves were observed on fewer than five leaves compared with 75% for roots—consistent with the occurrence of higher taxonomic turnover on aboveground plant compartments. Indeed, β-diversity analysis of Z. marina symbiont communities revealed major differences in above- versus belowground eelgrass microbiomes and their relationships with the surrounding environment (Fig. 1c and d). A permutational analysis of similarities (ANOSIM) of pairwise community Canberra distances revealed leaf communities with taxonomic compositions that strongly resembled those of seawater (*r* = 0.18, Benjamini-Hochberg adjusted *P* < 0.001) (Fig. 1c). By contrast, root communities were relatively similar to one another and were compositionally distinct from sediment (*r* = 0.69, *P* < 0.001; compare *r* statistics between significant relationships) (Fig. 1b).

The taxonomic compositions of leaves and seawater microbiomes were more similar within seagrass beds than between them (*P* = 0.005) (Fig. 1d, blue points), a result that is consistent with a leaf community driven by the microbial composition of the local ocean environment. By contrast, we did not detect a higher degree of compositional similarity between roots and sediment sampled from the same seagrass bed relative to other beds (*P* = 0.185) (Fig. 1d, red points), suggesting more homogenous root microbiome taxonomic compositions at the global scale. These results were recapitulated by a multivariate dispersion analysis (permdisp2 procedure) (29), which identified aboveground host and environmental community compositions that exhibited variances that were indistinguishable from one another (*P* = 0.96) and root microbiomes that were globally less variable compared with leaf communities (*P* < 0.001).

Analyses of rarefied Faith's phylogenetic diversity (PD) (30) and normalized unweighted UniFrac distances (31) revealed similar qualitative results for patterns in phylogenetic α- and β-diversities (Fig. 1e to g). An analysis of Faith's PD showed higher phylogenetic diversity (Fig. 1e) of root communities compared with those detected on eelgrass leaves (LME analysis of Faith's PD; *F*_3,311_ = 106.4, *P* < 0.001) when rarefied to a common sampling depth. We did not detect a difference in PD between eelgrass leaf and seawater microbial communities (*P* = 0.991), a result that recapitulates those from analyses of taxonomic community diversities (compare Fig. 1b and e). Likewise, a permutational analysis of similarities (ANOSIM) of pairwise community UniFrac distances revealed leaf communities that were relatively phylogenetically similar to microbial seawater communities (Fig. 1f) (*r* = 0.165, adjusted *P* < 0.001), whereas root communities were phylogenetically more distinct from sediment communities (*r* = 0.593, *P* < 0.001; compare *r* statistics). However, in contrast to results from taxonomic diversity analyses, eelgrass leaves were not more phylogenetically similar to seawater communities from the same seagrass bed in comparison to seawater communities from other seagrass beds, indicating that seawater harbors globally phylogenetically similar communities that can assemble onto seagrass leaves despite differences in the specific OTUs present in different locations.

### Environmental sources of eelgrass-associated microorganisms.

**FIG 2:**
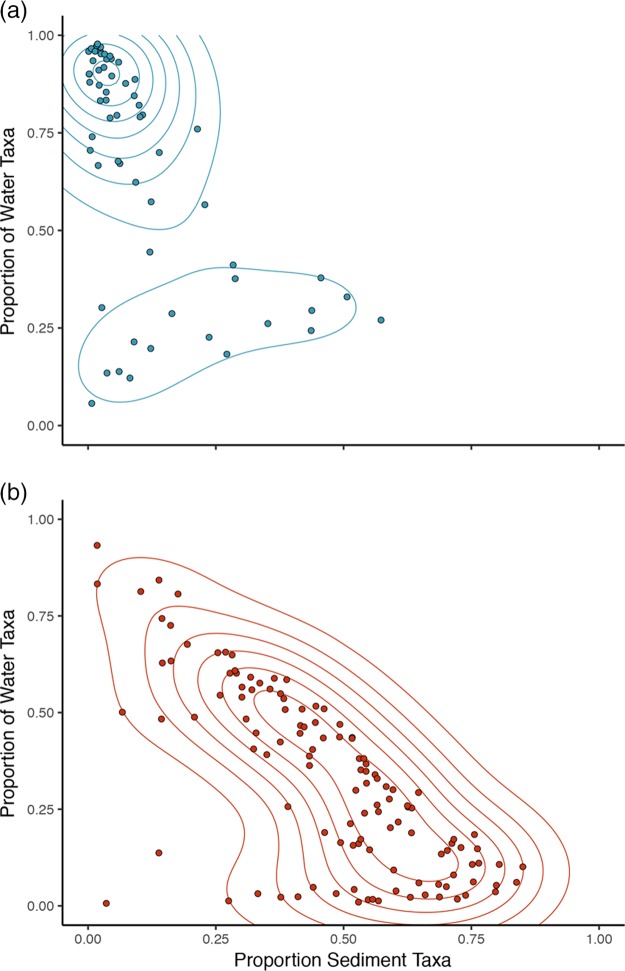
Results of SourceTracker analysis for leaf (a) and root (b) samples, where points represent individual microbial communities. Colors are the same as in Fig. 1: blue, leaves; and red, roots. Contours are drawn according to a 2-dimensional Gaussian kernel used for density estimation and delineate dense clusters of data points.

Environmental sources of microorganisms detected on seagrass leaves and roots were estimated by training a Bayesian source tracking classifier (SourceTracker [32]) on the set of seawater and sediment habitat samples to estimate the fraction of OTUs detected on each leaf and root that originated from these habitats. The model estimates that seawater is the primary source of colonists for seagrass leaves (median proportion of seawater-sourced OTUs, 0.8) (Fig. 2a), with many leaf samples appearing nearly entirely seawater sourced (Fig. 2a). Roots were estimated to be primarily sourced from sediment (median proportion of sediment-sourced OTUs, 0.51) (Fig. 2b). Although some root communities were predicted to originate nearly completely from sediments, most appeared to receive colonists from both above- and belowground environments (Fig. 2b).

**FIG 3:**
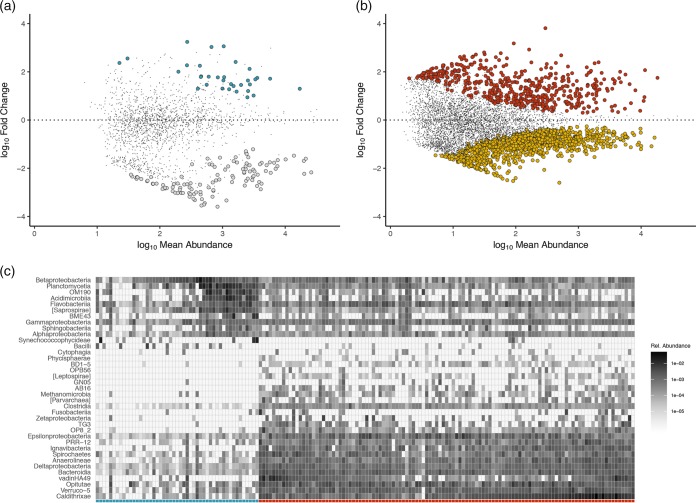
Host compartments are enriched and depleted for certain OTUs. (a) Enrichment and depletion of OTUs detected on leaves compared with those from the seawater environment as determined by differential abundance analysis. Each point represents an individual OTU, and the position along the *y* axis represents the abundance fold change relative to the primary source environment. Colors are the same as in Fig. 1; significantly enriched and depleted OTUs are blue and silver, respectively. (b) Results of differential abundance analysis for OTUs detected on roots compared with those from the sediment environment. Significantly enriched and depleted OTUs are red and gold, respectively. (c) Heatmap showing the taxonomic compositions of enriched taxa, aggregated at the class level, on leaves and roots. Darker shades of gray correspond to higher mean relative abundances of OTUs in each taxonomic class. Leaf and root samples are differentiated on the *x* axis by blue and red markers, respectively. White tiles indicate taxa that were not detected in particular samples. Matrix seriation (the process of rearranging matrix rows and columns for visual interpretation) was accomplished using a principal component analysis.

We used the estimates from source tracking to perform a guided differential abundance analysis for each of the two plant compartments (i.e., leaves and roots) to identify OTUs that were significantly enriched or depleted on hosts relative to their mean normalized abundance in the adjacent putative colonization source. We observed 33 enriched and 116 significantly depleted OTUs on Z. marina leaves relative to seawater (Fig. 3a), revealing an aboveground host compartment in which fewer than 8% of detected taxa exhibited patterns in normalized abundance that differed from those observed for seawater communities. Leaf-enriched taxa were largely represented by members of the Betaproteobacteria, Planctomycetia, OM190, and Acidimicrobiia classes (Fig. 3c, blue columns). By contrast, we detected 529 enriched and 1,004 depleted OTUs on seagrass roots (Fig. 3b), consistent with a higher degree of host recruitment and higher selectivity against particular environmental microorganisms on belowground seagrass tissues; 23.2% of taxa detected on roots exhibited patterns in normalized abundance that differed from those observed in sediments. Notably, 51 of these root-enriched OTUs (ca. 10%) clustered onto the genus Sulfurimonas (Epsilonproteobacteria), of which most of the cultured isolates are sulfide oxidizers (33). Moreover, 24.6% of root-enriched OTUs were members of the Desulfobulbaceae, Desulfovibrionaceae, Desulfuromonadaceae, or Desulfobacteraceae families or the Arcobacter genus, highlighting the acquisition of a diverse set of OTUs related to taxa involved in sulfur metabolism by belowground tissues as a potentially key process for marine angiosperms.

### Metabolic models of enriched taxa generate hypotheses about eelgrass microbiome assembly.

**FIG 4:**
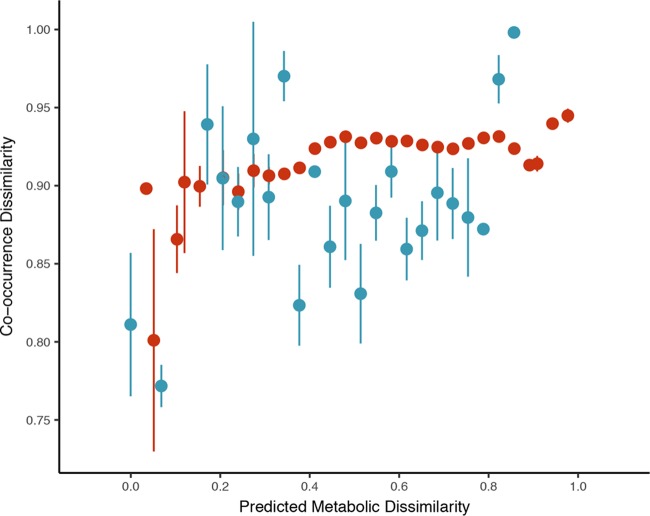
Relationships between OTU cooccurrence (Jaccard distances of rarefied OTU counts) and predicted metabolic dissimilarity (Jaccard distances between OTU seed sets) matrices for host-enriched taxa. To avoid the overplotting associated with the visualization of the more than 1.5 × 10^5^ pairwise comparisons, we visualized the relationships between matrices using binned scatterplots of mean Jaccard distances ± 2 SEM. Colors are the same as in Fig. 1: blue, leaves; and red, roots. Relationship between binned leaf-enriched OTU Jaccard distance and competitive dissimilarity matrices are highly variable and nonsignificant (*P* = 0.141). However, a significant positive relationship for root taxa (*P* = 0.008) is visible and consistent with a habitat-filtering community assembly mechanism (61).

Leaf- and root-enriched taxa exhibited median similarities to the 16S rRNA sequences of their most similar genomes of 92.0% and 92.9%, respectively. Imposing a minimum threshold of similarity to reference genome sequences of 94% or 97% similarity did not affect the qualitative predictions from metabolic modeling presented below. We did not detect a significant relationship between dissimilarity in predicted metabolic resource use and OTU cooccurrence dissimilarity for enriched taxa on leaves (Mantel test, *P* = 0.141) (Fig. 4). However, a significant positive relationship was observed among root-enriched OTUs (Mantel test, *P* = 0.008) (Fig. 4), suggesting that enriched belowground taxa that share a higher fraction of their predicted metabolic resources cooccur more frequently on root surfaces on average. Importantly, this relationship held when we accounted for pairwise phylogenetic branch lengths between OTUs (partial Mantel test, *P* = 0.009), suggesting that predictions from metabolic modeling were not simply recapitulating the phylogenetic relationships between taxa (34).

## DISCUSSION

Our global study of the Zostera marina eelgrass microbiome revealed a high degree of similarity between leaf and seawater communities in comparison to those of root surfaces, whose taxonomic and phylogenetic compositions were less heterogeneous than, and more distinct from, the surrounding sediment (Fig. 1). As very few studies describe the structure of microbiomes associated with aquatic plant surfaces compared with those of terrestrial species (35), the observations of terrestrial plants serve as an important reference. Our results identify notable contrasts in the structure of the eelgrass microbiome compared with those observed on well-studied terrestrial species. For instance, the similarity between seagrass leaf and adjacent seawater microbiome compositions differs from relationships observed for terrestrial plant leaves, which appear distinct from the microbial communities observed in air samples (36–39). Eelgrass leaves in our study exhibited microbiome compositions that strongly mirrored their surrounding seawater communities (Fig. 1c). Notably, Z. marina has lost genes for the production of volatile terpenes and lacks stomata on leaves (20), raising the possibility that seagrass leaves lack many of the characteristics of terrestrial plants (e.g., localized gas exchange via stomata, chemical defense, and communication) thought to influence the structure of their associated leaf microbiomes.

The widespread success of seagrasses has occurred despite environmental challenges. In particular, organic matter accumulation within coastal sediments causes sediment sulfide conditions that are toxic for vascular plants (40–42). The most abundant of the root-enriched microbial taxa detected in this study clustered within the genus Sulfurimonas, which accounted for ca. 10% of all root-enriched OTUs. All but one of the previously isolated strains of Sulfurimonas can oxidize sulfide and produce sulfate as an end product, suggesting that the recruitment of these bacteria may be critical for host tolerance of coastal marine habitats. The oxidation of sulfide and its precipitation as nontoxic S^0^ on the host's aerenchymous tissue have previously been attributed to host detoxification mechanisms such as the leakage of oxygen from root tips (42). However, the enrichment of Epsilonproteobacteria such as Sulfurimonas on root surfaces and the consistency of this pattern at the global scale add support to the hypothesis that microbial symbioses with particular taxa facilitate seagrass hosts' management of abiotic conditions in coastal beds. Indeed, abundant bacteria that are predicted sulfur oxidizers have been detected in marine sediments attached to seagrass roots (27), and terminal restriction fragment length polymorphism (T-RFLP) community profiling (43) of root surfaces in a single European seagrass bed has suggested similar patterns in Epsilonproteobacteria community dominance (44). This result is also consistent with observations that Spartina cordgrasses in coastal salt marshes are associated with abundant sulfur-oxidizing bacteria (45).

Predictions from metabolic modeling analyses suggest that eelgrass hosts may enrich for these microorganisms in part via the supply of particular metabolic compounds to belowground plant compartments. But, while predictions from metabolic modeling are consistent with prior studies of host-supplied compounds on seagrass surfaces (e.g., see reference 23), these predictions come with caveats and should be interpreted as hypotheses. The metabolic models analyzed herein are derived from 16S rRNA sequences and involve automated metabolic network reconstruction (46, 47). This approach may be less accurate than manual curation of metabolic models and is limited by the availabilities of sequenced prokaryotic genomes, but it permits the analysis of a large number of microbial taxa that would otherwise be intractable and indeed reflects a large proportion of the metabolic needs of these organisms (47). Here, we emphasize that the predictions from metabolic modeling will need to be validated by further empirical study. For instance, these predictions could be evaluated using a shotgun metagenomic approach combined with mass spectrometry to probe the physiology of taxa, such as Sulfurimonas, that we suspect play functional roles in eelgrass' tolerance of marine sediment conditions and their numerical associations with host-derived metabolites detected on seagrass tissues.

Prior research has documented a positive relationship between seagrass biomass production rates and the density of sulfide-consuming lucinid clams in seagrass beds, owing to the hypothesized *in situ* oxidation of sulfide by symbiotic bacteria housed in clam gills (41). However, in a meta-analysis of temperate seagrass beds, only 50% of sampled beds contained lucinid bivalves, and clam density was low in these beds relative to that in tropical sites (41). Thus, unless sulfide concentrations are lower in temperate sediments, temperate seagrasses must either be more tolerant of sulfides or have alternative means of detoxification. Physiological host processes such as oxygen leakage from roots (42) certainly contribute to sulfide oxidation, but our data suggest a role for microorganisms directly associated with eelgrass surfaces; Sulfurimonas bacteria were detected on all but one root sample in our global data set. Therefore, experimental efforts are needed to quantify the magnitudes of sulfur metabolism from these disparate processes (oxygen leakage by eelgrasses, symbionts associated with lucinid bivalves, and eelgrass root-associated bacteria) under different biotic and abiotic conditions to uncover the relative contributions of host- versus mutualism-based strategies by vascular plants in marine sediments for tolerating toxic sulfide concentrations. Our results indicate that this will be an important enterprise for future research.

Seagrasses and their ecosystems have been the subject of a great amount of research covering topics including ecology and biogeography (48), evolution (49), physiology (19), and genetics (20). Here, we have provided a global-scale characterization of the microbial communities associated with Z. marina seagrasses by contrasting host samples with those of their surrounding environments across the entire Northern Hemisphere. We hope that this will encourage researchers to study the microbiomes of other plant hosts across their geographic ranges, as such large-scale studies produce the empirical knowledge needed to develop a deeper understanding of microbial roles in the ecology and evolution of plants and the ecosystems that depend on them.

## MATERIALS AND METHODS

Microbial communities were sampled by researchers in the Zostera Experimental Network (ZEN), a global network of seagrass scientists (e.g., see reference 17). Three leaf, root, water, and sediment samples were collected from each of two physically separated seagrass beds at 25 ZEN locations (50 seagrass beds total) (Fig. 1a) using identical sampling protocols, were placed into 2-ml collection vials, and were covered in ZYMO Xpedition buffer. Root and leaf samples were acquired by collecting 10 root hairs and a 2-cm section of healthy green outer leaf blade, respectively. Seawater samples were collected just above each plant by filtering approximately 300 ml of seawater through a 0.22-μm filter and retaining filters. Finally, 0.25 g of sediment was taken adjacent to roots from 1 cm under the surface using a syringe. All samples were sent to the University of California, Davis, for DNA extraction, library preparation, and sequencing.

Community DNA was extracted using a modified version of the Mo Bio PowerSoil DNA extraction kit experienced user protocol. Modifications were to remove the precipitate formed by the ZYMO buffer and C1 solution. Namely, tubes were incubated at 65°C for 5 min to remove the precipitate and then homogenized in a bead beater. Instead of eluting DNA in solution C6, we added 50 μl of sterile nuclease-free water to the membrane before storing at −20°C. Following a modified PCR protocol from the Earth Microbiome Project (50), we used the bacterial and archaeal primers 515F and 806R with an in-house dual barcode system (see reference 51) to enrich for the V4 region of the 16S rRNA gene. Cycling conditions were an initial denaturation at 94°C for 3 min and 35 cycles of 94°C for 45 s, 50°C for 60 s, and 72°C for 90 s. We used peptide nucleic acid (PNA) blockers to reduce chloroplast and mitochondrial sequence products (52), which used 1 to 5 μl of template DNA. PCR amplicons were purified with Axygen AxyPrep Mag PCR clean-up kits, quantified using Qubit, and pooled in equal concentrations of DNA. Libraries were sequenced on an Illumina MiSeq generating 250-bp paired-end reads.

Raw sequence data were processed with QIIME 1.9.1 (53) and clustered into OTUs at >97% similarity using the closed reference UCLUST algorithm (54) against the Greengene*s* version 13.8 database. To ensure adequate sampling depth for analyses of community diversities, we omitted several samples from our analyses because they contained fewer than 2,500 sequences after quality control, thereby retaining data from 118 unique plants in total. We also omitted all 16S rRNA sequences identified as chloroplasts or mitochondria from subsequent analyses. Finally, we removed three OTUs identified as putative contaminants that were highly abundant in negative controls (blank-template samples from the PowerSoil DNA extraction kit), using the methods described by Meadow et al. (30).

### Statistical analyses.

OTU counts were rarefied to a sequencing depth of 2,500 sequences per sample for diversity analyses. Community taxonomic and phylogenetic diversities (i.e., α diversities) were calculated at this sampling depth using the Shannon and Faith's PD (55) indices, respectively. We tested for relationships between community type (i.e., leaves, roots, water, or sediment) and diversity using LME model analyses. Namely, we fit a model of the form *Y = B*_1_(community type) *+ G + H + E*, where *Y* is the diversity index, *B*_1_ is a regression coefficient, *G* and *H* are random effects of the seagrass bed, to account for the fact that multiple plants were measured from the same bed, nested within a spatial block (ZEN location), and *E* is a vector of errors. The normality of residuals was confirmed in all models using quantile-quantile plots. All models were fitted using the nlme package in the statistical programming environment R (56). Pairwise contrasts were performed with the implementation of Tukey's *post hoc* test in the multcomp package.

Community taxonomic and phylogenetic compositional dissimilarities (i.e., β diversities) between host and environmental samples were calculated using the Canberra and normalized unweighted UniFrac (31) distance measures, respectively. Canberra distances were calculated using rarefied OTU counts, whereas the UniFrac measure quantifies dissimilarities between communities based on phylogenetic relationships between OTUs that are detected. Dissimilarities of host and environmental samples were visualized using an unconstrained principal-coordinate analysis (PCoA). Effect sizes of dissimilarities between seagrass microbial and environmental communities were quantified using a permutational analysis of similarities (ANOSIM), constraining matrix permutations to within ZEN locations to account for potential pseudoreplication (57) in the data set. Differences in group variances were tested using a multivariate homogeneity of group dispersion analysis (permdisp2 procedure [29]) with an analysis of variance (ANOVA) and Tukey's *post hoc* test for pairwise contrasts.

To test the hypothesis that eelgrass-associated microbiomes were more similar to their adjacent environmental communities than to others (i.e., within- versus between-bed comparisons), we analyzed community dissimilarities of host and environmental samples at the scale of the seagrass bed using a Monte Carlo bootstrapping approach following ordination (e.g., see reference 58). Specifically, we calculated the distances between group centroids of replicate host samples taken from the same seagrass bed and the centroids of their corresponding environmental samples in PCoA space. We then determined whether host-associated microbial communities were more similar to the microbial community in the adjacent environment than to that of all other seagrass beds by comparing intercentroid distances against the distributions generated from 1,000 permutations of the randomized data set. Performing β-diversity analyses for both the Canberra and UniFrac distance measures allowed us to determine the degree to which microbiomes found on different compartments of the same host differed from one another and from those of their surrounding environments, both compositionally and phylogenetically.

Environmental sources of microorganisms detected on seagrass leaves and roots were estimated by training a Bayesian source tracking classifier (SourceTracker [32]) on rarefied OTU counts from the sets of water and sediment microbiome communities from each sampled coastline before testing the model on corresponding host-associated communities. The model assumes that host communities comprise a combination of colonists that originated from known and unknown exogenous sources and, using a Bayesian approach, estimates the fraction of OTUs detected on each leaf and root surface that originated from water, sediment, or unknown habitats.

We used the estimates from this classifier to perform guided differential abundance analyses for the two host compartments to identify OTUs that were significantly enriched or depleted on leaves and roots relative to their primary putative colonization source. The unrarefied OTU feature table was normalized using the trimmed mean of M values (TMM) method (59), which was selected due to its improved sensitivity for detecting differentially abundant taxa (see below) compared with that by rarefaction (60). Generalized linear models with negative binomial error distributions were fitted to TMM-normalized OTU counts after removing underpowered OTUs (OTUs that were detected fewer than five times), and differentially abundant taxa on host samples were identified using a likelihood ratio test. We focused subsequent analyses on OTUs that were significantly host enriched (Benjamini-Hochberg adjusted *P* < 0.01), as these taxa represent portions of the microbiome that were most likely to be actively selected for by the host (61).

Potential drivers of the acquisition of enriched taxa were investigated using whole-genomic metabolic modeling (28, 34, 46, 47) of these taxa or their closest relatives with fully sequenced genomes in the NCBI reference database (62). Namely, we used these models to generate hypotheses about associations between predicted metabolic interactions among these organisms and patterns in their cooccurrence or exclusion. Details of this analysis are discussed in references 28, 34, and 47, but briefly, we conducted a BLAST sequence similarity search (63) comparing each enriched OTU to a database of 16S rRNA gene amplicon sequences for prokaryotic taxa with whole-genome sequences in NCBI, compiled by Mendes-Soares et al. (47). The ModelSEED framework (46) was used to reconstruct and gap-fill metabolic models for the genomes most similar to eelgrass-enriched OTUs. Models were represented as topological networks where nodes denote chemical compounds and directed edges connect reactants to products. Using these networks, each OTU's seed set (sensu 28)—the minimal set of compounds an organism exogenously acquires to synthesize all others in its metabolic network—was calculated as a proxy for its resource profile (34) using a previously published graph-theoretic method (28). After computing each enriched OTU's seed set, a competitive dissimilarity matrix, C, was generated, which contained elements, *c_ij_*, representing the Jaccard distances between the seed sets of OTUs *i* and *j*. The relationship between cooccurrence dissimilarity (measured as Jaccard distances of OTU abundance profiles) and OTU resource dissimilarity (C) matrices were assessed for leaf- and root-enriched taxa using Mantel tests with 1,000 matrix permutations. If enriched taxa are more likely to cooccur when they utilize similar predicted metabolic resources, then we would expect a positive correlation between the cooccurrence and C dissimilarity matrices. Such patterns are consistent with a community assembly mechanism whereby organisms that require a particular set of resources tend to cooccur in environments that contain those resources (34).

### Accession number(s).

The 16S rRNA gene sequences from this project have been deposited in the sequence read archive (SRA) under accession number PRJNA379026.
